# Supplementary material for: Chicken swarm optimization modelling for cognitive radio networks using deep belief network-enabled spectrum sensing technique
Source: PLoS One. 2024 Aug 8;19(8):e0305987. doi: 10.1371/journal.pone.0305987 (PMC11309510; doi:10.1371/journal.pone.0305987)
Supplement: Attachment — Submitted filename: Final review comments with response.docx [file pone.0305987.s001.docx]

**Reviewer #1**: 1. Incoherence in Text: Sections 2.1 and 2.2 of the paper identify some limitations of existing spectrum sensing techniques, such as sensing time, algorithm complexity, and regularization constraints. However, these limitations are not further discussed or addressed in the subsequent text. It is recommended that the article adopt a structure that separates the overview from the details to enhance coherence.

**Response:** Thanks for your valuable suggestion. We agree for the above comments. Additionally, we have included sensing time, algorithm complexity, and regularization constraints in revised manuscript to make them clear and easy to view. Refer page no 18.

One crucial parameter, sensing time, highlights how well the model can sense and adjust to dynamic changes in the radio environment. For quick decision-making and resource allocation, the least amount of sensing time must be achieved. Conversely, algorithmic complexity is a computational issue that needs to be handled with caution. It is crucial for scalability and practicality to strike a balance between the computing demands of DBN and the complexities of CSO during training and inference. Additionally, regularization requirements are essential for guaranteeing the generalization of the model and avoiding overfitting. The robust performance of the model across various CRN scenarios depends on finding the ideal balance between exploration and exploitation inside the CSO framework while respecting regularization limitations.

2.Lack of Innovation: The abstract and introduction provide limited information about the innovation, which seems to be a simple combination of existing CSA algorithm and DBN.

**Response:** Thanks for your valuable suggestion, we have added detail description about abstract and introduction in the revised manuscript. Refer Page No 1-5.

3.Confusion in Explanation: For example, the definitions of signal modification and noise alteration in Equation (3) are unclear and confusing. The definitions of H1 and H0 in (4) and (5) are not provided. The full name of RBM is not explained. The definitions of variables N and T on Page 12 are repeated and inconsistent with those on Page 8. The simulation does not provide any introduction for the benchmarks RMLSSCRN-100 and RMLSSCRN-300.

**Response:** Thanks for your valuable suggestion. We agree for the above comments. We have modified equation 3, explanation for T, N, H0 and H1 in the revised manuscript. Refer page no 10 & 11. Additionally, we have added explanation for RMLSSCRN in the result and discussion section to make them clear and easy to view.

$H_{0}: Y\left( n \right)=W\left( n \right), :Primary user absent$	(1)

$$H_{1}:Y\left( n \right)=S\left( n \right)+W\left( n \right), :Primary user present$$

Where $n=1,2,3,....,N$is the example number of the sampled signal that has been received, $Y\left(n\right)$is the sampled signal that secondary users have received, $W\left(n\right)$ is the noise presented by the AWGN channel with zero mean and variance $\sigma_{n}^{2}$ .$s\left(n\right)$ is the signal from PU with variance $\sigma_{s}^{2}$ and zero mean, and h is the channel's impulse response or the channel amplitude gain between the PU transmitter and secondary user (SU) receiver since we use AWGN channel $h=1$.. $H_{0}$and $H_{1}$stand for the alternative hypothesis (existence of the PU) and absence (null hypothesis), respectively.

$H_{1}: T\sim Normal ((\sigma_{n}^{2}+\sigma_{s}^{2}), 2N \left( \sigma_{n}^{2}+\sigma_{s}^{2})^{2} \right)$	(3)

In the equation, $\sigma_{s}^{2}$&$\sigma_{n}^{2}$ represent the signal modification and noise alteration, correspondingly. Where N is the sample number, T stands for test statistics is related to the inception $\left( \lambda\right)$ for making the last decision on whether the primary signal has been current. The efficiency of energy detectors is considered three parameters existing according to the test measurements below the binary hypotheses.

4.Lengthy Simulation with Insufficient Depth: The simulation provides a mechanical description of the experiment results without in-depth analysis. It is recommended to integrate the simulation results with the previous introduction for a more comprehensive analysis.

**Response:** Thanks for your valuable suggestion, change has been made in the revised paper to make them clear and easy to view. Refer page no 20.

**Reviewer #2:** 1- I suggest to the authors that a native speaker review the manuscript to ensure the quality of the writing and the spelling.

**Response:** Thanks for your valuable suggestion, change has been made in the revised paper. to make them clear and easy to view.

2- The abstract lacks clarity and focus, requiring incorporating the underlying motivation behind the proposed methods.

**Response:** Thanks for your valuable suggestion, we have changed abstract part in the revised manuscript. Refer Page No 1.

In addition, the online spectrum sensing is carried out during the actual communication phase to adapt to dynamic changes in the spectrum environment in real-time. Offline spectrum sensing is typically performed during a dedicated sensing period before actual communication begins. The goal is to gather information about the spectral environment in advance. When combined with DBN's deep learning capabilities and CSO's innate nature-inspired algorithms, a synergistic framework is created that enables CRNs to explore and allocate frequencies on their own with astonishing accuracy. The parameters such as probability of detection, SNR of -24dB, the SST-CRN perfect has increased a developed Pd of 0.810, whereas the RMLSSCRN-100 and RMLSSCRN-300 methods have accomplished a lower Pd of 0.577 and 0.736, respectively.

3- Explicitly specifying the paper's novelty and highlighting its advancements in relation to recent state-of-the-art studies within a similar domain would add value.

**Response:** Thanks for your valuable suggestion, we have added novelty and highlighting its advancements in relation to recent state-of-the-art studies in introduction part in the revised manuscript. Refer Page No 2-4.

4- A wide range of statistical analysis and comparison with other techniques are essential.

**Response:** Thanks for your valuable suggestion. We agree for the above comments. Additionally, we have included statistical analysis in the literature survey section in revised manuscript to make them clear and easy to view.

**Table 1. A summary of related work in CRN**

| **Authors** | **Year** | **Techniques** | **Gaps or concept not covered** |
| --- | --- | --- | --- |
| A. Fawzi et al. [22] | 2022 | Two methods ED and WD are combined to propose two-stage spectrum sensing strategies. | The Shannon entropy is the only topic of this paper. It excludes Tsallis, Renyi, and Kapur entropy, among other forms of entropy. |
| A. D. Sahithi et al. [23] | 2022 | To address the issue of fading and hidden primary terminal uncertainty, cooperative spectrum sensing with ED is employed. | The suggested approach is not resistant to noise improbability at low SNR, while improving SS performance. Furthermore, there is a significant increase in sensing time and computing complexity since WD and ED are merged at low SNR. |
| G. Prieto et al. [24] | 2019 | A detection technique for spectrum sensing based on entropy is suggested. A number of guidelines for figuring out how many bins a histogram has are assessed. Here are those guidelines: Scott rule, Sturges rule, and square root rule | The goal of this work is not to solve the noise uncertainty issue. |
| F. Mashta et al. [25] | 2021 | The discussion of two- and three-stage SS detectors is done in detail.  Utilizing various smoothing factors, ED and maximum eigenvalue detector are employed. | Because of its sensitivity to noise uncertainty, SS performs poorly overall at low SNR even with three- and two-stage spectrum sensing. Furthermore, the system's total complexity rises with the employment of the eigenvalue-based detector. |

5- The paper could benefit from providing more detailed information about the hyperparameter selection process. Including the range of values explored, the chosen values, and the rationale behind the selection would enhance the transparency of the experimental setup and allow for better reproducibility.

Response: Thanks for your valuable suggestion. We have added hyperparameter selection process. Including the range of values explored, the chosen values, and the rationale behind the selection in the revised manuscript. Refer proposed section 3.1.

**Fig 3. Flowchart of CSA**

6- The paper lacks details on the implementation and availability of codes. It would greatly benefit readers and future researchers to have access to the codes to replicate and facilitate further exploration of the proposed method.

Response: Thanks for your valuable suggestion. We have added implementation and availability of codes in the revised manuscript. Refer proposed section.

**Pseudo code of CSO**

1: Prime the populace of chicken as a matrix k;

2: Determines the values of fitness for each row using the k variable;

3: While (t<gen)

4: t = t+1;

5: If (t %G == 0)

6:	According to their level of physical activity,

k should be split into three groups: the rooster, the hens, and the chicks.

7: else

8:	For i = 1:

9:	If (i = rooster);

Upgrade the position of rooster using Eq (20);

end if

10:	If (i = hen);

Upgrade the position of hen using Eq (21);

end if

11:	If (i = chick);

Upgrade the position of chick using Eq (25);

end if

12:	Upgrade the new explanation of k

13: end

14: end

7- How to run the program for the user-provided data? How to repeat the results described in the manuscript? I strongly recommend adding more detailed information, such as the saved model, the defined labels, data processing, etc. It is better to provide the documentation and sample files.

Response: Thanks for your valuable suggestion. We have added documentation and sample files in the revised manuscript.

Refer this link. https://github.com/sjjana77/deep-belief-network

8- It is better to provide an analysis of the reason for the model's excellent performance.

**Response:** In response to the reviewer's remarks, this issue has been addressed in the revised paper.

9- The manuscript contains a lot of data but lacks more figures to make the results more convincing.

**Response:** Thanks for your valuable suggestion. We agree for the above comments. We have added figures 3 and 4 in the revised manuscript to make them clear and easy to view. Refer proposed method.

10- The conclusion did not summarize the improvement of the approach over other approaches and highlight what limitations the system solves. Therefore, the authors need to explain why this method has improved significantly.

Response: Thanks for your valuable suggestion. We have highlighted limitations to solve the proposed method and quantitative results in the revised manuscript. Refer conclusion section.

The limitations of proposed CSO-DBN model presents an additional degree of complexity in terms of the difficulty of reaching optimal parameter tuning because the deep learning network's parameters interact with the optimization algorithm's parameters, requiring precise calibration. Furthermore, it is still difficult to make sure the model can adapt to dynamic and unpredictable CRN environments, particularly when network circumstances are changing quickly. Scalability issues also come up, especially in large-scale CRNs where the optimization process may not be as efficient. The presented model can be tested on various SS approaches as part of the future scope. Additionally, mathematical equations can be optimized to decrease computational density and progress detection performance.

11- The authors describe the methods used in detail. However, this is more literature information. It would enhance the quality of the paper if more information about the implementation and algorithms of the novel workflow were provided, too.

**Response:** Thanks for your valuable suggestion. We agree for the above comments. We have added flowchart and pseudo code in the revised manuscript.

12- The results section needs some language revision to make it more readable.

**Response:** In response to the reviewer's remarks, this issue has been addressed in the revised paper.

13- You need to provide the final form of the overall loss function in the text and explain how you are weighting the different components and deciding on those weights.

**Response:** Thanks for your valuable suggestion. We agree for the above comments. We have added overall loss function in CSO in the revised manuscript.

All of the comparative tests in this study are conducted using Python 3.8.5 in a PC with 8GB of RAM [31]. Let us assume that the population size is 50, the maximum number of iterations is 500, the α in the fitness function is set to 0.9999, and there are 20 independent running experiments on the datasets. The selection scheme corresponding to each particle is tested for classification accuracy using the classifier.

14- Check that all of your Figures and Tables explain your text well.

**Response:** In response to the reviewer's remarks, this issue has been addressed in the revised paper.

15- In this paper, the advantages and novelty of the proposed scheme are not given.

**Response:** Thanks for your valuable suggestion, we have added novelty and advantages of proposed method in the revised manuscript. Refer introduction section.

Energy detection is a SS model that depends on determining the absence/presence of the primary user and measuring the established signal energy by relating the selected energy level with inception. Extensive research was conducted in the survey to find the optimum threshold expression and also to develop SS performances. The researchers projected a novel technique for adaptive threshold selection in multi-band detection. Evaluating the threshold can be implemented by the function of the primary and secondary indicators of the established signals. The detection performance could be evaluated using two metrics: detection probability (DP) represents the chance that a CR user will report a PU is available when the spectra are actually being used by a PU, and false alarm probability (FA) represents the chance that a CR user will report a PU is available while the spectra are empty. Since a detection miss would demand PU intervention and an FA would lower the SE, it is typically necessary for optimal detection performance in situations where the chance of detection is increasingly susceptible to an FA probability. The effectiveness of detection in SS can be severely restricted by a number of issues, including multipath fading, shadowing, and receiver uncertainty.

16- It is difficult to know how to implement the proposed scheme.

Lastly, I want to emphasize that the availability of a reproducible source code. Without these components, I regret to say that I would recommend rejecting the paper.

**Response:** In response to the reviewer's remarks, this issue has been addressed in the revised paper.

Refer this link. https://github.com/sjjana77/deep-belief-network

**Reviewer #3**: The abstract provides a clear overview of the research on a new spectral sensing technique for cognitive radio networks (SST-CRN). Here are some suggestions:

Clarity and Conciseness:
The abstract is generally clear and concise, which is great. However, it might benefit from a slight restructuring for improved flow and readability.

**Response:** Thanks for your valuable suggestion, we have changed abstract part in the revised manuscript. Refer Page No 1.

In addition, the online spectrum sensing is carried out during the actual communication phase to adapt to dynamic changes in the spectrum environment in real-time. Offline spectrum sensing is typically performed during a dedicated sensing period before actual communication begins. The goal is to gather information about the spectral environment in advance. When combined with DBN's deep learning capabilities and CSO's innate nature-inspired algorithms, a synergistic framework is created that enables CRNs to explore and allocate frequencies on their own with astonishing accuracy. The parameters such as probability of detection, SNR of -24dB, the SST-CRN perfect has increased a developed Pd of 0.810, whereas the RMLSSCRN-100 and RMLSSCRN-300 methods have accomplished a lower Pd of 0.577 and 0.736, respectively.

Acronyms and Terminology:
Ensure that acronyms like CRN, QoS, SNR, CSA, and DBN are defined upon first use to aid readers who may not be familiar with the specific terms.

**Response:** In response to the reviewer's remarks, this issue has been addressed in the revised paper.

Quality of Service (QoS)

Signal to Noise Ratio (SNR)

Cognitive Radio Networks (CRN)

Cyclo-stationary Feature Detection (CFD)

Restricted Boltzmann machine (RBM)

Introduction of the Problem:
Consider expanding the introduction to clearly highlight the challenges faced in spectrum sensing in cognitive radio networks. This could help set the stage for the proposed solution.

**Response:** Thanks for your valuable suggestion, we have highlighted the challenges faced in spectrum sensing in cognitive radio networks in introduction part in the revised manuscript. Refer Page No 2-4.

Methodology Explanation:
While you briefly mention the chicken swarm algorithm (CSA) and deep belief network (DBN), providing a bit more detail about these elements in the abstract could enhance understanding for readers.

**Response:** Thanks for your valuable suggestion, we have added chicken swarm algorithm (CSA) and deep belief network (DBN) in abstract part in the revised manuscript. Refer Page No 1.

The goal is to gather information about the spectral environment in advance. When combined with DBN's deep learning capabilities and CSO's innate nature-inspired algorithms, a synergistic framework is created that enables CRNs to explore and allocate frequencies on their own with astonishing accuracy. The parameters such as probability of detection, SNR of -24dB, the SST-CRN perfect has increased a developed Pd of 0.810, whereas the RMLSSCRN-100 and RMLSSCRN-300 methods have accomplished a lower Pd of 0.577 and 0.736, respectively.

Offline and Online Operations:
The abstract mentions the two stages of operations (offline and online), but it might be beneficial to briefly elaborate on what each stage entails. It could help readers grasp the methodology more comprehensively.

**Response:** Thanks for your valuable suggestion, we have done offline and online operations in abstract part in the revised manuscript. Refer Page No 1.

online spectrum sensing is carried out during the actual communication phase to adapt to dynamic changes in the spectrum environment in real-time. Offline spectrum sensing is typically performed during a dedicated sensing period before actual communication begins. The goal is to gather information about the spectral environment in advance

Performance Validation:
Include a sentence or two on the specific measures or metrics used for performance validation and highlight the superiority of the proposed model. It would provide a glimpse into the results and attract more interest.

**Response:** Thanks for your valuable suggestion. We agree for the above comments. We have added metrics used for performance validation in section 4.

Language and Style:
Consider avoiding passive voice where possible. For instance, instead of saying "The performance validation process pointed out," you might say "Performance validation results demonstrated."

**Response:** Thanks for your valuable suggestion, change has been made in the revised paper to make them clear and easy to view.

The performance validation results demonstrated the authority of the projected model and terminated current state-of-the-art techniques in standings of various measures.

Clarity and Structure:
The introduction is informative but could benefit from a clearer structure. Consider breaking it down into smaller paragraphs to enhance readability.

**Response:** In response to the reviewer's remarks, this issue has been addressed in the revised paper.

Definition of Terms:
While terms like spectrum sensing (SS), cyclo-stationary feature detection, and energy detection are mentioned, providing brief definitions or explanations for these terms could help readers unfamiliar with the specific terminology.

**Response:** Thanks for your valuable suggestion, we have done definitions of cyclo-stationary feature detection, and energy detection in introduction part in the revised manuscript. Refer Page No 3.

Cyclo-stationary Feature Detection (CFD) is the technique of recognizing and evaluating cyclo-stationary characteristics in a signal. Certain types of signals exhibit cyclo-stationarity, which means that their statistical features change periodically across time. Energy Detection (ED) is commonly used for SS due to its noncoherent approach, which does not require prior knowledge of PU signals for the SU receiver to operate. Its sensing time is fast and its processing complexity is low. In wireless communication systems, noise uncertainty a random and inevitable fluctuation in noise significantly reduces ED performance, mainly at low SNR.

Transition to the Proposed Model:
The transition to the proposed model (SST-CRN) in the later part of the introduction is well-executed. However, consider providing a sentence that explicitly states the gap or problem in the existing methods that the proposed model aims to address.

**Response:** Thanks for your valuable suggestion. We agree for the above comments. We have added research gap in section 1 in the revised manuscript. Refer page no 4.

One significant gap is the limited examination of the model's applicability to highly dynamic and varied CRN environments. Existing research frequently falls short of fully addressing the issues provided by abrupt changes in spectrum circumstances, such as fluctuating interference levels and dynamic channel occupancy. Furthermore, there is a gap in the investigation of the model's performance at various network scales, with minimal attention paid to scaling concerns in large-scale CRNs. Furthermore, nuanced research of the influence of communication overhead within the CSO swarm intelligence framework, particularly in the case of large networks, is still unexplored area. Bridging these gaps is critical for increasing the efficacy and real-world usability of CSO-DBN models in the complex and dynamic environments of Cognitive Radio Networks.

Spectrum Sensing Techniques:
The description of various spectrum sensing techniques is informative, but it might be beneficial to briefly explain why certain techniques are preferred in specific scenarios. This could provide context for readers less familiar with the field.

**Response:** In response to the reviewer's remarks, this issue has been addressed in the revised paper.

Focus on Energy Detection:
The detailed explanation of energy detection and its challenges is well-presented. However, consider briefly summarizing the main challenges before introducing the proposed model.

**Response:** Thanks for your valuable suggestion. We agree for the above comments. We have added main challenges before introducing the proposed model in the revised manuscript. Refer page no 8.

Simulation Analyses:
It's mentioned that a wide range of simulation analyses was performed, but the abstract doesn't provide a glimpse into the results. Consider including a sentence that hints at the findings or the superiority of the proposed SST-CRN technique.

**Response:** Thanks for your valuable suggestion, we have done numerical achievement in abstract part in the revised manuscript. Refer Page No 1.

Section Organization:
The outline of the remaining sections (literature study, constraints, problem identification, modeling, reproduction findings, and conclusion) is clear. However, consider rephrasing to make it more concise.

**Response:** Thanks for your valuable suggestion. We agree for the above comments. Additionally, we have rephrased the whole manuscript to make them clear and easy to view.

Spectrum has long been the focus of intensive research since it is an important component in wireless communication systems. Potential cognitive radio technology employs white spaces to enable spectrum detection and opportunistic spectrum consumption. Given the challenges faced by cognitive radios, adopting a spectrum sensing strategy appears to be critical to getting good outcomes in terms of properly employing the available spectrum while minimizing interference with licensed leading operators. This work introduces a new SST-CRN approach to improve SS efficiency in CRN. The SST-CRN technique aids in the finding of nonlinear thresholds based on the CSA-DBN model, in which the DBN model's parameters are ideally selected using the CSA. The suggested SST-CRN approach works in two stages: offline and online. A wide range of simulation analyses were accepted, with the results examined in several ways. When DBN's deep learning capabilities are paired with CSO's natural nature-inspired algorithms, a synergistic framework is formed that allows CRNs to explore and allocate frequencies independently with astounding accuracy. The factors such as likelihood of detection, SNR of -24dB, and the SST-CRN perfect have enhanced the developed Pd of 0.810, whereas the RMLSSCRN-100 and RMLSSCRN-300 methods have achieved a lesser Pd of 0.577 and 0.736, respectively. The parameters such as probability of error analysis with -SNR of -24dB, the SST-CRN technique has resulted in a lower Pe of 0.066 when compared to the existing model.

**Figure 1 has been change****d**
