# Supplementary material for: Chicken swarm optimization modelling for cognitive radio networks using deep belief network-enabled spectrum sensing technique
Source: PLoS One. 2024 Aug 8;19(8):e0305987. doi: 10.1371/journal.pone.0305987 (PMC11309510; doi:10.1371/journal.pone.0305987)
Supplement: Attachment — Submitted filename: Final review comments with response SST-CRN 26.4.2024.docx [file pone.0305987.s002.docx]

Reviewer #3:

The abstract effectively introduces the problem of spectrum sensing in CRNs and outlines the proposed solution. However, you could rephrase some sentences for clarity and conciseness. For example, the phrase "The proposed SST-CRN technique involves two stages of operations, namely offline and online" could be simplified to enhance readability.
The abstract presents numerical results indicating the performance of SST-CRN in terms of Pd under specific conditions (e.g., SNR of -24dB). While these results provide valuable insights, it would be beneficial to include a brief interpretation or discussion of these results to underscore the effectiveness of the proposed technique.
It could be valuable to conclude the abstract by briefly mentioning potential future directions or areas for further research, such as scalability, robustness in dynamic environments, or real-world deployment considerations.

**Response:** Thanks for your valuable suggestion. We agree for the above comments. We have added detailed description about the abstract part in the revised manuscript to make them clear and easy to view. Refer page no 2.

Cognitive radio networks (CRN) enable wireless devices to sense the radio spectrum, determine the frequency state channels, and reconfigure the communication variables to satisfy Quality of Service (QoS) needs by reducing energy utilization. In CRN, spectrum sensing is an essential process that is highly challenging and can be addressed by several traditional techniques, such as energy detection, match filtering, etc. At the same time, the performance of the existing models gets affected by the low Signal to Noise Ratio (SNR) of received signals and the minimum amount of received signal samples. To resolve the drawbacks of traditional energy detection models, this paper presents a new spectral sensing technique for cognitive radio networks (SST-CRN). The proposed model helps obtain a nonlinear threshold based on a chicken swarm algorithm (CSA) with a deep belief network (DBN). The proposed DBN enabled SST-CRN technique undergo a structured process comprising two distinct stages: offline and online. In the offline stage, the DBN model is rigorously trained on pre-collected data, learning to extract complicated patterns and representations from the radio environment's spectral characteristics. This phase entails extensive feature extraction, model optimization, and validation to assure the DBN's ability to capture complex spectral dynamics. In addition, the online spectrum sensing is carried out during the actual communication phase to adapt to dynamic changes in the spectrum environment in real-time. Offline spectrum sensing is typically performed during a dedicated sensing period before actual communication begins. When combined with DBN's deep learning capabilities and CSO's innate nature-inspired algorithms, a synergistic framework is created that enables CRNs to explore and allocate frequencies on their own with astonishing accuracy. The proposed solution significantly improves the spectrum efficiency and resilience of CRNs by harnessing the power of DBN, which leads to more effective resource utilization and less interference. The Simulation results show that our proposed strategy produces more accurate spectrum occupancy assessments. The result parameters such as probability of detection, SNR of -24dB, the SST-CRN perfect has increased a developed Pd of 0.810, whereas the existing methods RMLSSCRN-100 and RMLSSCRN-300 have accomplished a lower Pd of 0.577 and 0.736, respectively. Our deep learning methodology uses convolutional neural networks to automatically learn and adapt to dynamic and complicated radio environments, improving accuracy and flexibility over classic spectrum sensing approaches. Future research might focus on improving CSO algorithms to better optimize the spectrum sensing process, enhancing the reliability of DBN-enabled sensing techniques.

For introduction:
The paper mentions limited research on highly dynamic CRN environments and network scaling. However, a more specific discussion on how the proposed method addresses these gaps would strengthen the argument.

**Response:** Thanks for your valuable suggestion. We have added detailed description about research gap and strengthen in the revised manuscript to make them clear and easy to view

A potential research gap in the existing method of SST-CRN approach based on the DBN-Enabled spectrum sensing technique is the investigation of robustness and scalability. While the current strategy shows potential in improving spectrum sensing performance using deep learning techniques, further research is needed to determine its adaptability to different and dynamic CRN situations. Research might focus on analyzing the technique's performance in contexts with heterogeneous network topologies, changing interference levels, and dynamic user behaviors. Addressing the scalability issues associated with implementing DBN-based solutions in large-scale CRN deployments is another significant topic for investigation. Investigating techniques to maximize computational resources, model training efficiency, and inference speed has the potential to considerably improve the proposed technique's practical applicability and deployment feasibility in real-world CRN scenarios. To overcome this research gap would help to further our understanding and practical application of DBN-enabled SST-CRN allowing for more reliable and efficient spectrum utilization.

While the paper acknowledges the need to explore communication overhead within the CSA framework, a dedicated section analyzing its impact, particularly in large-scale networks, is necessary.

**Response:** Thanks for your valuable suggestion, change has been made in the revised paper to make them clear and easy to view

Recognizing the crucial role that communication overhead plays in the performance and scalability of optimization algorithms by thoroughly examining communication overhead factors such as message passing, data exchange, and synchronization mechanisms, particularly in the context of large-scale CRNs, this section aims to provide valuable insights into the efficiency and feasibility of using CSA for spectrum optimization. This investigation aims to provide realistic ideas and tactics for addressing communication overhead concerns, hence increasing the applicability and scalability of CSA-based optimization techniques in real-world CRN implementations.

The review mentions positive simulation results but lacks specifics. Including key metrics like detection probability, false alarm probability, and spectrum efficiency achieved by SST-CRN compared to established methods would solidify the claims of its effectiveness.

**Response:** Thanks for your valuable suggestion, change has been made in the revised paper to make them clear and easy to view

For literature review:
While summarizing existing techniques is helpful, you can include a brief comparison of their advantages and disadvantages.
Briefly explain how the reviewed methods relate to the proposed SST-CRN technique (Chicken Swarm Optimization with Deep Belief Network). Are they competing approaches, complementary methods, or foundational techniques upon which SST-CRN builds?

Response: Thanks for your valuable suggestion. We agree for the above comments change has been made in the literature section to make clear and easy to view. Refer Page no 7 to 9.

**Table 1. A summary of related work in CRN**

| **Authors** | **Year** | **Techniques** | **Gaps or concept not covered** | **Advantages** | **Disadvantages** |
| --- | --- | --- | --- | --- | --- |
| A. Fawzi et al. [21] | 2022 | Two methods ED and WD are combined to propose two-stage spectrum sensing strategies. | The Shannon entropy is the only topic of this paper. It excludes Tsallis, Renyi, and Kapur entropy, among other forms of entropy. | - Easy to implement - No prior knowledge of the primary signal characteristics is required | - High false alarm rate - Unreliable at low SNR values - Sensitive to noise uncertainty |
| A. D. Sahithi et al. [22] | 2022 | To address the issue of fading and hidden primary terminal uncertainty, cooperative spectrum sensing with ED is employed. | The suggested approach is not resistant to noise improbability at low SNR, while improving SS performance. Furthermore, there is a significant increase in sensing time and computing complexity since WD and ED are merged at low SNR. | - Robust against noise uncertainty - Distinguish between signal and noise - Decreased probability of false alarm at low SNR | - Large sensing time to achieve a good performance - High energy consumption when the size of the samples is large |
| G. Prieto et al. [23] | 2019 | A detection technique for spectrum sensing based on entropy is suggested. A number of guidelines for figuring out how many bins a histogram has are assessed. Here are those guidelines: Scott rule, Sturges rule, and square root rule | The goal of this work is not to solve the noise uncertainty issue. | - Better detection at low SNR region - Optimal sensing | - Prior knowledge of the primary user signal is required - Impractical since prior knowledge about the signal is not always available |
| F. Mashta et al. [24] | 2021 | The discussion of two- and three-stage SS detectors is done in detail.  Utilizing various smoothing factors, ED and maximum eigenvalue detector are employed. | Because of its sensitivity to noise uncertainty, SS performs poorly overall at low SNR even with three- and two-stage spectrum sensing. Furthermore, the system's total complexity rises with the employment of the eigenvalue-based detector. | - Machine learning can detect if trained correctly can be a good approach - Minimize the delay of the detection | - Complex techniques - Has to be adapted in learning in very fast changing environments - Features selection affects detection rate and adds complexity |

The existing methodologies in CRNs frequently struggle to discover dynamic spectrum possibilities in complicated and noisy situations, resulting in inefficient spectrum usage. In contrast, the proposed SST-CRN method represents a considerable divergence from existing approaches by incorporating DBNs into the spectrum sensing process. While most existing methods rely on simple signal identification algorithms, the proposed strategy uses deep learning to extract complicated patterns and representations from high-dimensional spectrum data. The proposed strategy uses DBNs to overcome the limits of traditional methods by improving spectrum sensing accuracy, flexibility, and robustness in CRNs. Thus, while previous methods set the framework for spectrum sensing in CRNs, the proposed strategy represents a transformative step forward by offering a novel approach that leverages deep learning capabilities to enable more efficient and reliable spectrum utilization.

For the proposed model:
The section lacks information on the model's performance. Including simulation results or comparisons with existing methods would strengthen the analysis.

**Response:** Thanks for your valuable suggestion. We agree for the above comments. We have added performance of simulations results in the revised manuscript to make clear and easy to view.

**Probability of detection:**

The possibility that a cognitive radio system can accurately detect the existence of a primary user signal through background noise or interference, permitting effective use of available spectrum resources, is known as the probability of detection.

$$P_{d}=Q(\sqrt{\frac{2\gamma}{N_{0}}})$$

Where:

𝑄 ( 𝑥 ) standard normal distribution's complementary cumulative distribution function is denoted as Q(x).

γ is the SNR of the received signal.

The noise power spectral density is denoted by 𝑁 = 0.

**Pnf :** Probability non-fading channels refers to the possibility of meeting wireless communication channels that do not exhibit considerable fading over time during the spectrum sensing process of a cognitive radio network. This method entails finding and utilizing available spectrum opportunities in stable channels in order to improve the efficiency and reliability of spectrum usage in cognitive radio networks.

**Pmd :** The probability of miss alarm (Pmd) in Cognitive Radio Networks Enabled SST depicts the possibility of failing to identify the attendance of a primary user signal when it is present, indicating a potential inefficiency in spectrum sensing in cognitive radio systems.

$$P_{miss}=1-\Pi_{i=1}^{N}P_{\det ection_{i}}$$

Where:

$P_{miss}$ the likelihood of missing an alert, or going unreported, is denoted as P (miss).

N represents the total number of CR users in the network.

$P_{\det ection_{i}}$ Detection I is the likelihood of detection for the i-th CR user.

**Probability of error:** The probability of error detection is the possibility that this technique will correctly identify communication-ready spectrum bands while reducing the possibility of missing or misdetecting signals, which is essential for effective spectrum use and dependable network performance.

$$P_{e}=1-\Pi_{i=1}^{N}(1-P_{e_{i}})$$

$P_{e}$ is the total likelihood of error in the SST-CRN model with multiple CR users.

$P_{e_{i}}$ is the probability of error for the i-th CR user.

Briefly discuss the potential limitations of the proposed model. For example, you can mention the computational expenses involved in training the Deep Belief Network (DBN) and the potential risk of overfitting.

**Response:** Thanks for your valuable suggestion. We agree for the above comments. We have added potential limitations of the proposed model in the revised manuscript to make clear and easy to view. Refer result and discussion section.

The proposed SST-CRN methods show promise for improving spectrum sensing in CRNs, but it also faces potential limitations. First challenge is the computational complexity of training and deploying deep learning models such as DBNs, which may limit real-time implementation, particularly in resource-constrained CRN devices. Furthermore, the effectiveness of the suggested technique may be significantly reliant on the availability and quality of training data, which may be limited or biased, thus compromising the model's generalization capabilities in varied CRN contexts. Furthermore, the interpretability of DBN-based spectrum sensing judgments may be problematic, making it difficult to explain and confirm the reasons behind certain detection results. Finally, while DBNs excel at learning complicated patterns, they may struggle in contexts with fast changing dynamics or adversarial conditions, requiring constant adaptation and refining to remain effective. Addressing these restrictions is critical to realizing the suggested model's full potential in real-world CRN implementations.

For Result and Discussion:
The results demonstrate that the SST-CRN model offers superior performance in spectrum sensing compared to the benchmark methods under non-fading and fading channel conditions. However, the analysis lacks information on the underlying functionalities of these benchmark methods.

**Response:** Thanks for your valuable suggestion. We agree for the above comments. We have added benchmark method RMLSSCRN-100 and RMLSSCRN-300 in the revised manuscript to make clear and easy to view. Refer result and discussion section.

The benchmark approaches RMLSSCRN-100 and RMLSSCRN-300 possess certain essential features that enhance their efficiency in spectrum sensing in Cognitive Radio Networks (CRNs). Both methods utilize Random Matrix Learning-based Spectrum Sensing (RMLSS) methodologies, which exploit principles from random matrix theory and machine learning algorithms. These approaches facilitate the distinction between noise and signal components in the spectrum, which is essential for precise spectrum sensing. In addition, RMLSSCRN-100 and RMLSSCRN-300 utilize sophisticated algorithms and specialized feature sets to improve the accuracy and reliability of spectrum detection. These algorithms probably include preprocessing processes to enhance raw input data, feature selection approaches to discover important spectral properties, and model modifications to enhance overall performance. Moreover, these methods may use statistical features of incoming signals, such as eigenvalue decomposition and hypothesis testing, to aid in making spectrum sensing decisions. In general, RMLSSCRN-100 and RMLSSCRN-300 exhibit advanced features designed to enhance spectrum sensing in CRNs, making them valuable standards for comparing with other approaches.

For Conclusion
It restates specific results from the previous section without a broader context.
The conclusion avoids using technical jargon introduced earlier (Pd, Pe, Pnf).
It also doesn't offer a concluding statement on the overall effectiveness of the model.

Response: Thanks for your valuable suggestion. We agree for the above comments change has been made in the conclusion section to make clear and easy to view. Refer conclusion section.

As an important component of cognitive radio, spectrum sensing is also a key technique for improving the efficiency of spectrum utilization and achieving reasonable and efficient allocation of spectrum resources, which has important research value and application prospects. While traditional spectrum-sensing algorithms no longer satisfy the increasingly complex sensing environment, researchers continue to incorporate novel approaches such as deep learning and deep reinforcement learning to address emerging problems in this area. In this study presents an enhancing the performance of CRN the integration of CSO, a bio-inspired optimization algorithm inspired by the collective behavior of chickens, with DBN-enabled spectrum sensing, this approach offers a novel solution for addressing the spectrum scarcity problem and improving spectrum utilization efficiency in CRNs. By leveraging CSO, the proposed model optimizes the deployment of cognitive radios and the allocation of spectrum resources, enabling CRNs to adapt dynamically to changing environmental conditions and user demands. Furthermore, the integration of DBN-based spectrum sensing enhances the cognitive capabilities of CRNs by enabling accurate and adaptive spectrum sensing in complex and dynamic radio environments. The synergistic combination of CSO modeling and DBN-enabled spectrum sensing demonstrates significant potential for enhancing the reliability, efficiency, and scalability of CRNs, ultimately facilitating the realization of more intelligent and autonomous wireless communication systems. The SST-CRN technique aids in the finding of nonlinear thresholds based on the CSA-DBN model, in which the DBN model's parameters are ideally selected using the CSA. A wide range of simulation analyses were accepted, with the results examined in several ways. When DBN's deep learning capabilities are paired with CSO's natural nature-inspired algorithms, a synergistic framework is formed that allows CRNs to explore and allocate frequencies independently with astounding accuracy. The factors such as likelihood of detection, SNR of -24dB, and the SST-CRN perfect have enhanced the developed probability of detection of 0.810, whereas the RMLSSCRN-100 and RMLSSCRN-300 methods have achieved a lesser probability of detection of 0.577 and 0.736, respectively. The parameters such as probability of error analysis with SNR of -24dB, the SST-CRN technique has resulted in a lower probability of error of 0.066 when compared to the existing model. Future research should explore the application of extended reality technologies and artificial intelligence-based spectrum sensing, clustering, and energy harvesting in digital twin-based virtual factories that are backed by cognitive radio-based Internet of Things (IoT) networks.

Recommendations:
Briefly summarize the key performance improvements achieved by SST-CRN compared to existing methods.

**Response:** Thanks for your valuable suggestion, change has been made in the revised paper to make them clear and easy to view

The SST-CRN introduces significant performance improvements compared to existing methods. Firstly, it improves spectrum sensing accuracy by exploiting the deep learning capabilities of Deep Belief Networks (DBNs) to record complicated spectral patterns and dynamics. This leads to more accurate detection of available spectrum opportunities, maximizing spectral efficiency in CRNs. Furthermore, the suggested technique improves resilience to changing environmental circumstances and noise levels by continuous learning and adaption of DBN parameters. This adaptability increases the robustness of spectrum sensing, allowing for greater performance in dynamic and uncertain CRN situations. Furthermore, by utilizing unsupervised learning methods, the technique decreases the requirement for labeled training data, increasing scalability and application in real-world scenarios.

Reiterate the benefits of the DBN-CSO combination without reintroducing technical terms.

**Response:** Thanks for your valuable suggestion, change has been made in the revised paper to make them clear and easy to view

The combination of DBN with CSO provides various benefits. First, it improves spectrum sensing accuracy by accurately recording complicated spectral patterns. This results in more reliable detection of accessible spectrum opportunities, hence increasing efficiency in Cognitive Radio Networks (CRNs). Second, the DBN-CSO combination improves flexibility to changing environmental conditions, ensuring strong performance in dynamic CRN situations.

Conclude by emphasizing the model's potential for improved spectrum utilization in CRNs while acknowledging the limitations for further research.

**Response:** Thanks for your valuable suggestion, change has been made in the revised paper to make them clear and easy to view

Future research should explore the application of extended reality technologies and artificial intelligence-based spectrum sensing, clustering, and energy harvesting in digital twin-based virtual factories that are backed by cognitive radio-based Internet of Things (IoT) networks.
