# Supplementary material for: Chicken swarm optimization modelling for cognitive radio networks using deep belief network-enabled spectrum sensing technique
Source: PLoS One. 2024 Aug 8;19(8):e0305987. doi: 10.1371/journal.pone.0305987 (PMC11309510; doi:10.1371/journal.pone.0305987)
Supplement: Attachment — Submitted filename: Final Review comments with response.docx [file pone.0305987.s003.docx]

**Comments to the Author**

1. If the authors have adequately addressed your comments raised in a previous round of review and you feel that this manuscript is now acceptable for publication, you may indicate that here to bypass the “Comments to the Author” section, enter your conflict of interest statement in the “Confidential to Editor” section, and submit your "Accept" recommendation.

Reviewer #3: (No Response)

2. Is the manuscript technically sound, and do the data support the conclusions?

The manuscript must describe a technically sound piece of scientific research with data that supports the conclusions. Experiments must have been conducted rigorously, with appropriate controls, replication, and sample sizes. The conclusions must be drawn appropriately based on the data presented.

Reviewer #3: Yes

3. Has the statistical analysis been performed appropriately and rigorously?

Reviewer #3: Yes

4. Have the authors made all data underlying the findings in their manuscript fully available?

The [PLOS Data policy](http://www.plosone.org/static/policies.action#sharing) requires authors to make all data underlying the findings described in their manuscript fully available without restriction, with rare exception (please refer to the Data Availability Statement in the manuscript PDF file). The data should be provided as part of the manuscript or its supporting information, or deposited to a public repository. For example, in addition to summary statistics, the data points behind means, medians and variance measures should be available. If there are restrictions on publicly sharing data—e.g. participant privacy or use of data from a third party—those must be specified.

Reviewer #3: Yes

5. Is the manuscript presented in an intelligible fashion and written in standard English?

PLOS ONE does not copyedit accepted manuscripts, so the language in submitted articles must be clear, correct, and unambiguous. Any typographical or grammatical errors should be corrected at revision, so please note any specific errors here.

Reviewer #3: Yes

6. Review Comments to the Author

Please use the space provided to explain your answers to the questions above. You may also include additional comments for the author, including concerns about dual publication, research ethics, or publication ethics. (Please upload your review as an attachment if it exceeds 20,000 characters)

Reviewer #3: Thank you for your revision. Most of the suggestions has been addressed. However there are few suggestion which I think will improve the quality of the paper:

. Introduction:
Please clearly define the research problem and objectives in the introduction to provide a more focused direction for the study.

**Response:** Thanks for your valuable suggestion, we have added research problem and objectives in the introduction section to make them clear and easy to view.

The existing methods for SST-CSO modeling in Cognitive Radio Networks (CRNs) that employ Deep Belief Network (DBN)-enabled spectrum sensing encounter problems such as large computational overhead and slow convergence speeds. These difficulties impede real-time application and efficiency in dynamic spectrum environments. Furthermore, the complexity of training DBNs and their susceptibility to parameter adjustment hinder their implementation, resulting in inefficient spectrum sensing and resource allocation in heterogeneous radio frequency environments. The proposed SST-CRN method efforts to improve the efficiency and accuracy of spectrum sensing, a fundamental task in CRNs, by exploiting CSO's powerful optimization capabilities. The integration with DBNs aims to take advantage of deep learning's ability to recognize subtle patterns and correlations in the radio frequency spectrum, which will improve channel identification. Furthermore, this hybrid method aims to lower the computational complexity and energy consumption associated with classic spectrum sensing techniques, hence increasing the operational lifespan of CRNs.

2. Related Work:
Provide a critical analysis of the strengths and weaknesses of each reviewed technique.
Include a comparative analysis to highlight the advantages of CSO and DBN over existing methods.

**Response:** Thanks for your valuable suggestion. We agree for the above comments. We have added strengths and weaknesses of existing system in the revised manuscript to make them clear and easy to view. Refer Literature section.

Compared to conventional optimization methods, CSO provides higher convergence rates and precision in high-dimensional search spaces, which makes it ideal for CRNs because of their complex and dynamic structure. This method, when combined with DBNs, makes use of deep learning's capacity to recognize complex relationships and patterns in spectrum data, resulting in more accurate and dependable spectrum sensing. In addition to lowering the computational load, this hybrid approach improves the overall resource allocation and utilization in CRNs by effectively balancing exploration and exploitation during optimization. Better resilience to non-stationary and heterogeneous radio environments is another feature of the CSO-DBN paradigm that guarantees more stable and interference-resistant communication. Overall, the CSO-DBN technique addresses the drawbacks of conventional approaches and satisfies the expanding requirements of contemporary wireless networks by offering a more intelligent, flexible, and effective solution for spectrum management.

3. Methodology:
It lacks clarity in explaining the selection criteria for parameters such as population size and maximum iterations. So. clearly explain the rationale behind parameter selection to justify their choices. Also, provide more detailed explanations of the CSO and DBN algorithms for readers unfamiliar with these techniques.

**Response:** Thanks for your valuable suggestion, change has been made in the revised paper to make them clear and easy to view.

To achieve optimal performance, it is important to set parameters for CSO in CRN using Deep Belief Network (DBN)-enabled spectrum sensing. In CSO, the population size must strike a balance between computing efficiency and diversity. A population size of 20–50 is often selected to allow for a thorough search space exploration while maintaining reasonable processing needs. Another crucial parameter is the maximum number of iterations, which is often set between 100 and 500 iterations to provide the algorithm enough time to converge to an ideal solution without creating undue delay a crucial factor in contexts with dynamic spectrum. Furthermore, CSO-specific characteristics, including the quantity of leaders and followers, are set up to preserve a balance between the search space's exploration and exploitation. The complexity of the CRN environment and the available computational resources are taken into consideration when fine-tuning these settings, which are tested empirically. The meticulous adjustment of parameters guarantees that the CSO algorithm combines with the DBN in an efficient and precise manner for spectrum sensing, hence improving the overall performance of the network.

Chicken Swarm Optimization (CSO) is a nature-inspired optimization algorithm modeled after the social behaviours and hierarchical structures of chicken swarms. In CSO, the population consists of roosters, hens, and chicks, each playing a distinct role in the optimization process. Roosters, representing the best solutions, guide the hens and chicks, which represent other candidate solutions. The algorithm iterates through movements and interactions among these groups to explore and exploit the search space effectively, aiming to find the optimal solution. Deep Belief Networks (DBNs) are a type of deep learning model composed of multiple layers of Restricted Boltzmann Machines (RBMs), which are simple, two-layer neural networks. DBNs are trained in a greedy layer-wise manner, starting with the first RBM and progressively training subsequent layers while keeping the previously trained layers fixed. This hierarchical learning approach allows DBNs to capture complex patterns and dependencies in data. In spectrum sensing for Cognitive Radio Networks (CRNs), DBNs are used to process and analyse the radio frequency spectrum data, identifying available channels with high accuracy. By combining CSO and DBN, we leverage CSO's optimization capabilities to fine-tune the parameters of the DBN, enhancing its performance in spectrum sensing. This synergy ensures efficient spectrum utilization and robust detection of available channels in dynamic wireless environments.

4. Results and Discussion:
Provide detailed analysis of the factors influencing the performance metrics, such as the impact of SNR levels on detection probabilities.
Discuss the implications of the results in terms of practical applications and theoretical advancements.

**Response:** Thanks for your valuable suggestion. We agree for the above comments. We have added performance metrics, such as the impact of SNR levels on detection probabilities in the revised manuscript to make them clear and easy to view and also included implications of the results in terms of practical applications and theoretical advancements in the revised manuscript.

The impact of SNR values on detection probability in spectrum sensing is significant. At high SNR levels, the primary user's signal is significantly stronger than the noise, resulting in higher detection probability and lower false alarm rates due to the clear separation between signal and noise. In contrast, at low SNR levels, the signal is closer in magnitude to the noise, making it harder to discern between the two. This leads to reduced detection probabilities and greater false alarm rates since the sensing algorithm fails to effectively recognize the presence of the primary user among the noise. The performance of spectrum sensing techniques is heavily dependent on SNR levels, with high SNR resulting in higher detection probability and low SNR providing considerable obstacles. Other important elements that influence detection performance include sensing time, channel conditions, noise characteristics, and signal qualities. Optimizing these aspects using modern algorithms and adaptive sensing approaches can considerably improve the reliability and efficiency of spectrum sensing in cognitive radio networks.

In practically, it leads to more efficient, reliable, and adaptable spectrum sensing in cognitive radio networks, which improves overall communication quality and device performance. Theoretically, it stimulates the development of sophisticated algorithms and models that push the limits of present spectrum sensing capabilities, driving innovation and growth in wireless communications.

5. Conclusion:
Include a discussion on the limitations of the proposed method, such as computational complexity and real-world implementation challenges.
Provide specific directions for future research to address the identified limitations and further advance the proposed approach.

**Response:** Thanks for your valuable suggestion, we have added detail description about limitations of proposed method and future research in the revised manuscript.

The major limitation of the proposed method SST-CRN is the high computational complexity and resource requirements of training and deploying DBNs, which can be difficult in situations when resources are limited or real-time. Furthermore, despite its effectiveness in optimization, the CSO algorithm can occasionally experience problems with convergence speed and become stuck in local optima, particularly in contexts with a highly dynamic and heterogeneous spectrum.

In Future research could focus on creating more efficient and scalable versions of CSO in order to improve its convergence features and reduce computational overhead. To address local optima issues, hybrid approaches combining CSO with other optimization techniques may be investigated. Furthermore, advances in lightweight and real-time deep learning models may make DBN-enabled spectrum sensing more useful for CRNs. Adaptive learning techniques could also be investigated, allowing DBNs to continuously learn and adapt to different spectrum settings with less input. Finally, incorporating modern data fusion algorithms to collect and process a variety of spectrum sensing data sources could increase the technique's dependability and accuracy.

6. References:
Ensure all references include complete publication details, including journal names, volume, issue, and page numbers.
Verify the accuracy of all references to maintain the quality of the citation list.

**Response:** Thanks for your valuable suggestion. We agree for the above comments. We have added below references in the revised manuscript to make them clear and easy to view. Refer Literature section.
